# Visually Evoked Potentials after Panretinal Photocoagulation in Omani Patients with Uncontrolled Diabetes Mellitus

**DOI:** 10.4103/0974-9233.51992

**Published:** 2008

**Authors:** Radha Shenoy, Habiba Al-Belushi, Sadiqa Al-Ajmi, Susan Margaret Al-Nabhani, Shyam Sunder Ganguly, Alexander A. Bialasiewicz

**Affiliations:** 1 From the Department of Ophthalmology, Sultan Qaboos University College of Medicine, Muscat, Al-Khod, Oman; 2 From the Department of Clinical Physiology, Sultan Qaboos University College of Medicine, Muscat, Al-Khod, Oman; 3 From the Department of Epidemiology and Medical Statistics, Sultan Qaboos University College of Medicine, Muscat, Al-Khod, Oman; 4 From the Department of Magrabi Eye and Ear Center, Al-Ahli Hospital, Doha, Qatar

**Keywords:** VEP, diabetes, laser

## Abstract

**Aim:**

To report on the changes of latency and amplitudes of the pattern VEP in patients with uncontrolled diabetes mellitus II and I before and after panretinal laser treatment.

**Design:**

Single center hospital based comparative study.

**Methods:**

One hundred eyes of patients with proliferative diabetic vitreoretinopathy, and HbA1C ≥ 10 percent were subjected to Pattern Visually Evoked Potentials (Medtronic keyopint system, Nicolet) prior to and 4 weeks after PRP. Results were compared to age-matched non-diabetic controls. Chi-Square test, and paired ‘t’ test were used for statistical analysis.

**Results:**

Preoperative mean VEP amplitude was 8.35mV±3.71, and not significantly different to the control group (mean 10.51mV±3.34) (chi square test p=1). Mean preoperative P100 latency was 106.93±7.90ms and significantly different to the control group (103.21±7.65ms) (paired t-test p=0.001). After laser treatment, VEP amplitudes decreased in 48/100 eyes (mean total 5.11mV±2.4), and P100 latency increased in 75/100 eyes (mean total 110.47±7.35ms).

**Conclusion:**

In this study, PRP was followed by a significant decrease in VEP amplitudes in 48 percent and increase in latency in 75 percent of eyes.

Diabetes mellitus can seriously impair electrophysiologycal and psychophysical parameters.^1^–^6^ Functional changes in the neurosensory system occur before any structural abnormalities become visible, and they may be reversible with a strict treatment regimen.^7^ Changes have been found to increase with time, age of patients, and the progression of retinopathy.^1^,^3^,^8^–^11^

While many studies have applied VEPs on controlled or treated diabetic patients, only few are available on persistently hyperglycemic ones with high HbA_1_C values. It has been recently shown in animal models that uncontrolled hyperglycemia is associated with specific biochemical degenerative changes in the neurosensory layer resulting in apoptosis of the neuroganglia.^12^–^16^

While the analysis of changes in the spectral components of the VEP has helped to develop an improved staging of diabetic retinopathy, the traditional role of destructive argon laser photocoagulation to stabilize the retinal disease has only recently been challenged. In this study we assess the VEP changes in a unique population of the Middle East with permanently uncontrolled diabetes mellitus and high HbA1C values in order to evaluate VEPs pre- and postoperatively.

## Methods

### Patients:

One hundred eyes of patients with proliferative diabetic retinopathy as defined by American Academy of Ophthalmology were selected for the study.

Included were patients with best corrected visual acuity of +1.0 logMAR and better, clear optic media and no evidence or history of other ocular diseases, and no history of surgery or laser. HbA_1_C ranged from 10 to 13% with a mean of 10%, and post prandial blood glucose examinations was 9.5 to 27 mmol/l with a mean of 14 mmol/l.

Excluded were patients with best corrected visual acuity of less than +1.0 logMAR, and cataract patients with nuclear opalescence of more than grade 3, nuclear color of more than grade 3 and cortical cataract of more than grade 3 (LOCS III classification).

All patients underwent a detailed ophthalmological examination, including fluorescein angiography.

Biomicroscopical presence or absence of maculopathy was noted and qualitatively assessed.*

The function of the central visual pathway was evaluated by pattern VEP. The results were compared to age-matched non-diabetic controls.

Optical coherence tomography (OCT) of the macula was performed in most patients, however, in this retrospective study the OCT was not consistently available in the files, and it was therefore not correlated with the other investigations.

Each study eye received argon laser treatment as recommended by the ETDRS. In patients who had both proliferative changes and maculopathy, the latter was dealt with focal laser coagulation initially followed by panretinal laser photocoagulation. Panretinal photocoagulation was applied for proliferative retinopathy.

### Panretinal Photocoagulation:

The eye receiving treatment was anesthetized with 1 drop of 0.4% Novesine (Oxybuprocain-HCL 4mg) and laser spots were delivered through a panfundoscope applied to the anesthetized eye. Total effects of 2500-3000 were applied with a spot diameter of 200-300µm, varying power 150-400 mW, and a burn time of 0.1-0.2 seconds. A scatter pattern of delivery was implemented extending from the posterior fundus to cover the peripheral retina in two to three sessions. The amount of treatment per session depended on patient pain threshold levels, with 400-500 spots delivered per session on an average.

### Pattern VEP:

Full field pattern VEP recordings were performed in a darkened, sound-attenuated room for electrodiagnostic procedures. The patient was seated one meter away from the pattern-shift screen At this viewing distance the check edges subtended 15 minutes of visual angle and the screen of the monitor subtended 12.5°. The visual stimuli were checkerboard patterns (contrast 70%, mean luminance 110cd/m2) generated on a TV monitor and reversed in contrast at the rate of two reversals per second. The stimulation was monocular, with occlusion of the contralateral eye.

Standard silver-silver chloride disc surface electrodes were fixed in the following positions: active electrode at Oz, reference electrode at Fpz, ground on the left ear (according to the international 10/20 electrode system). The interelectrode resistance was kept below 3kω. The bioelectric signal was amplified (gain 20000), filtered (bandpass, 1–100Hz), and averaged (200 events free from artifacts were averaged for every trial) with sweep speed 50ms/div and sensitivity 2μV/div using Nicolet Viking IV NT machine. The analysis time was 500ms intervals following a stimulus.

Two responses were recorded. About 100-200 stimuli per response were presented in each trial to ensure reproducibility in order to give a P100 latency within 2.5ms difference and a peak to peak amplitude of N75 P100 within a 15% difference. In cases of low amplitude responses, the stimuli were increased to 400 per response to ensure reproducibility. The central 6-12 degrees were evaluated using check size (24-32). Records were analyzed to identify the major components described in normal individuals like N75, P100, and N145. The P100 latency and amplitude was measured from baseline. If the P100 amplitude was low in occipital regions, additional testing was done by placing the recording electrodes at additional midline sites to detect the occurrence of the P100 peak maximally at occipital sites more rostral or caudal than the mid-occipital position.

In this study, patient visual acuity, fundus fluorescein angiography and VEP was repeated 6 weeks after laser treatment. The latency and amplitude in the study eye was compared to that from age-matched non-diabetic controls and compared later with results after photocoagulation therapy. Chi-Square test, and paired ‘t’ test were used for statistical analysis.

## Results

### Gender and Age:

The male:female ratio was 2:1 (67 males, 33 females), mean age was 53.65±9.39 years (age distribution: ≤40:2, 41-50:41, 51-60:26, 61-70:24, ≥71:7).

### Types and duration of diabetes mellitus:

**Figure 1:**
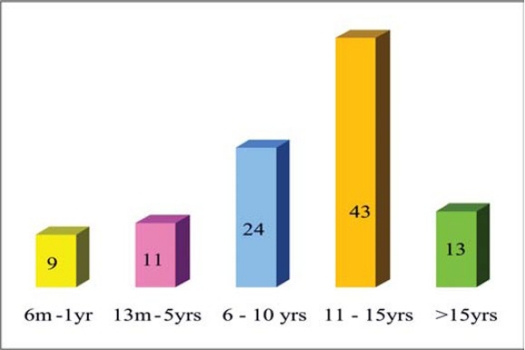
Diabetic age distribution in study group

Type II diabetes was more common than type I (ratio 15:1). Diabetes duration was 6 months to 22 years wit a mean of 6.4±4.9 years (distribution: ≤12 months: 9, 13 months to 5 years: 11, 6 to 10 years: 24, 11 to 15 years: 43, >15 years: 13). 6 of the 13 patients who had a disease duration of more than 15 years had type I diabetes (Fig 1).

### Retinopathy:

42/100 eyes in the study group had a diabetic maculopathy, {(mild 13 (31.0%) moderate 16 (38.1%) and severe 13 (31.0%)}, 18/100 eyes had preretinal gliosis along the arcades and paracentral area, and 40/100 eyes had proliferative changes without clinical or angiographic evidence of any maculopathy.

### Best Corrected Preoperative Visual Acuity and Morphology (Table 1):

**Table 1:** Visual Acuity Distribution Control and Study Eyes Pre-laser and Post-laser

VA log MAR	Control Eyes	PDR + no maculopathy		PDR + mild mac		PDR + mod mac		PDR + sev.macl		PDR + pre retinal gliosis		Total Eyes	
							
		Pre	Post	Pre	Post	Pre	Post	Pre	Post	Pre	Post	Pre	Post
1.1	0	0	0	0	0	0	0	0	0	0	4	0	4

1.0	40	17	16	6	3	7	2	10	7	18	14	58	42

0.8-0.7	37	12	11	3	6	4	9	3	6	0	0	22	32

0.6-0.4	19	5	7	3	3	5	5	0	0	0	0	13	15

0.3	4	6	6	1	1	0	0	0	0	0	0	7	7

Total	100	40	40	13	13	16	16	13	13	18	18	100	100

1. +1.0 logMAR in 58 eyes (17 eyes: PDVR without maculopathy, 6 eyes with mild, 7 moderate, and 10 with severe maculopathy, preretinal gliosis: 18 eyes).
2. 0.8-0.7 logMAR in 23 eyes (12 eyes: PDVR without maculopathy, 4 eyes with mild, 4 moderate, and 3 with severe maculopathy, preretinal gliosis: 0 eyes)
3. 0.6-0.4 logMAR in 13 eyes (5 eyes: PDVR without maculopathy, 3 eyes with mild, 5 moderate, and 0 with severe maculopathy, preretinal gliosis: 0 eyes)
4. ≤0.3 logMAR in 6 eyes (PDVR without maculopathy)

### Best Corrected Postoperative Visual Ccuity and Morphology (Table 1):

1. > +1.0 logMAR in 4 eyes (4 eyes: PDVR without maculopathy)
2. 1.0 logMAR in 42 eyes (16 eyes: PDVR without maculopathy, 2 eyes with mild, 2 moderate, and 7 with severe maculopathy, preretinal gliosis: 14 eyes) 2. 0.8-0.7 logMAR in 32 eyes (11 eyes: PDVR without maculopathy, 6 eyes with mild, 9 moderate, and 6 with severe maculopathy, preretinal gliosis: 0 eyes)
3. 0.6-0.4 logMAR in 15 eyes (7 eyes: PDVR without maculopathy, 3 eyes with mild, 5 moderate, and 0 with severe maculopathy, preretinal gliosis: 0 eyes)
4. ≤0.3 logMAR in 7 eyes (6 eyes: PDVR without maculopathy, 1 eyes with mild maculopathy)

### VEP Results:

Two out of 18 eyes with preretinal gliosis (11.1%) and 8 out of 13 eyes with PDVR and severe maculopathy (61.5%) showed no response to stimulation with LED goggles. Latency and amplitude was evaluated in the study eye and in the control eye and compared after eliminating the eyes that showed no response from the study eye group. Post laser amplitudes and P100 values were also evaluated in the study eye and compared with that in the prelaser period.

### Preoperative Amplitude:

The amplitude was considered normal if ≥6 mV, low if <6mV and absent if there was no VEP response.

There was no statistically significant difference between the mean amplitude in the study eyes (8.35µV±3.71) and in the control eyes (10.51µV±3.34) (chi square test p=1). Ten eyes in the study group showed no response to stimulation with LED goggles, 32 eyes had low amplitudes, and 58 eyes had normal amplitudes compared to 27 eyes with low amplitudes and 73 eyes with normal amplitudes in the control group.

A VEP could be recorded in all eyes of the control group compared to 10 eyes in the study group where no VEP could be recorded (chi-square test p <0.01) (Fig 2A).

**Figure 2A:**
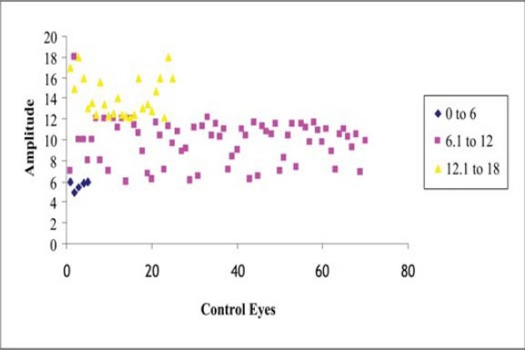
Visually evoked potentials in control eyes.

### Preoperative Amplitudes and Morphology (Fig. 2B):

**Figure 2B:**
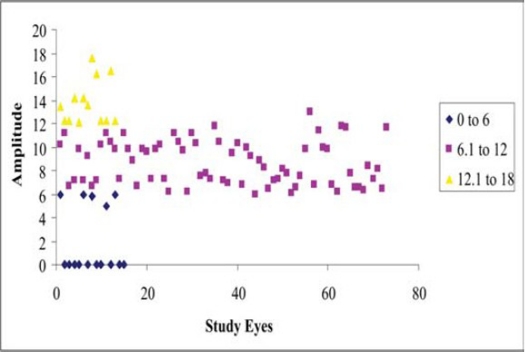
Visually evoked potentials in study eyes (pre-laser).

1. normal amplitude (58 eyes): 34 eyes: PDVR without maculopathy, and 11 with mild, 7 with moderate and 4 with severe maculopathy, 2 with epiretinal gliosis
2. low amplitude (32 eyes): 6 eyes: PDVR without maculopathy, and 2 with mild, 7 with moderate and 3 with severe maculopathy, 14 with epiretinal gliosis
3. absent amplitude (10 eyes): 2 eyes: PDVR with moderate, and 6 with severe maculopathy, preretinal gliosis: 2 eyes

### Postoperative Amplitudes:

40 eyes had a normal amplitude, 48 showed a low response, and 12 postoperative eyes (compared to 10 preoperative) showed no response (chi square test p=0.05). The eyes that recorded flat or no VEP had developed tractional retinal detachment. Thus, VEP amplitudes deteriorated in 18 eyes.

### Postoperative Amplitudes and Morphology (Fig. 2C):

**Figure 2C:**
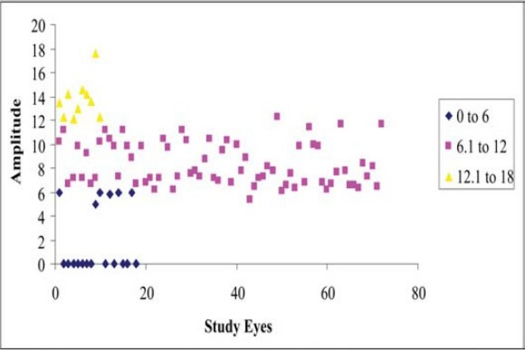
Visually evoked potentials amplitude in study eyes (post-laser).

1. normal amplitude (40 eyes): 32 eyes: PDVR without maculopathy, and 5 with mild, 5 with moderate and 1 with severe maculopathy, 0 with epiretinal gliosis
2. low amplitude (48 eyes): 8 eyes: PDVR without maculopathy, and 8 with mild, 12 with moderate and 6 with severe maculopathy, 14 with epiretinal gliosis
3. absent amplitudes (12 eyes): 2 eyes: PDVR with moderate and 6 with severe maculopathy, 4 with epiretinal gliosis

### Preoperative P100 Latency:

P100 latency was compared between study eyes and control eyes after excluding the patients with flat VEPs. 75/100 eyes showed an increased P100 latency, 47/75 eyes (62.7%) had a severe retinal disease with maculopathy and preretinal gliosis. Only 28/100 eyes in the control group had an increased P100 latency.

The mean P100 value was 106.93±7.9ms in the study group compared to 103.21±7.65ms in the control eyes. There was a statistically significant difference in the P100 values between the study eyes and the control eyes (paired ‘t’ test p= 0.001).

### Postoperative P100 Latency:

A statistically significant (paired ‘t’ test p<0.001) difference was noted between the mean preoperative (106.93±7.90ms) and postoperative (110.47±7.35ms) P100 values.

## Discussion

In this study we have shown increased functional impairment in uncontrolled diabetic patients undergoing laser treatment for proliferative diabetic vitreoretinopathy.

Functional impairment of the central nervous system is a frequent complication of diabetes mellitus, and may be due to multifactorial vascular and metabolic factors.^8^,^17^–^19^

The clinical importance in patients is discussed controversially. However, most previous studies have not characterized patients well with regard to their hyperglycemia, and there is a complete lack of observations in long-time and persistently high-level hyperglycemic patients declining medical treatment. Because it has been reported previously in animal models that different biochemical pathways exist in the uncontrolled hyperglycemic conditions, more pronounced morphological features may be expected in such a human population. Due to traditional beliefs and attitudes, decline of treatment for chronic diseases in general and diabetes specifically is a frequent feature of patients in the Middle East. Therefore, this study is unique.

VEPs measure the electrophysiological responses of the nervous system to visual stimuli. Abnormalities may present as changes in latency, amplitude, topography and wave form indicating clinically significant abnormalities. However, external factors, technical changes, patient cooperation, fixation, alertness, gender, age, media clarity, ocular blood flow and pupil size also produce apparent effects.^20^ Nevertheless, P100 latency prolongation is the most reliable indicator of clinically significant abnormality.

Significant reduction in amplitude and latency of the visual evoked responses at all spatial frequencies dissociated by the Snellen visual acuity measurements has been reported to occur in both types of diabetic patients without retinopathy, denoting a nonselective functional neuronal loss which probably preceded the ophthalmoscopically detectable features in these patients. Others noted similar responses in patients with varying grades of retinopathy, and noted the existence of a strong correlation with proliferative retinopathy and abnormal VEP which they attributed to extensive neuronal damage in the ganglion cell and nerve fiber layers of the retina in this group of patients.^1^,^10^,^17^,^21^–^27^

Similar finding were also noted in our study as well. A statistically significant number of preoperative study eyes already showed increased latency and some lower amplitudes. Furthermore, in the postoperative assessment the proportion of patients with increased latency was up by 75% and decreased amplitudes up by 47%. VEP amplitude mainly deteriorated in eyes with advanced disease and maculopathy. The VEP was absent in 10% of study eyes with advanced retinal disease. While an improvement of visual acuity in some eyes may have been due to the resolution of maculopathy it is not clear if the deterioration of VEP in these eyes, was due to the existence of systemic and local risk factors, or due to the fact that the argon laser photocoagulation stabilized only the peripheral (retina/ optic nerve) aspect of the disease while the central damage continued or that, it marked the cumulative effect of reduction in central and peripheral retinal sensitivities following full-scatter PRP to the existing central damage.^28^,^29^ Redistribution of neurochemicals in the visual cortex following pan retinal photo-coagulation, causing functional loss could also explain the deterioration of an already compromised visual system as was in our study.^30^

Diabetes mellitus has an effect on electrophysiological and psychophysical aspects of vision. The extent of mainly retinal destruction^27^ clearly has a role in promoting these abnormalities. However, it needs to be clarified if controlling the structural damage alone will help reduce the visual morbidity in this population.
